# Food Processing and the Mediterranean Diet

**DOI:** 10.3390/nu7095371

**Published:** 2015-09-17

**Authors:** Richard Hoffman, Mariette Gerber

**Affiliations:** 1 School of Life and Medical Sciences, University of Hertfordshire, Hatfield AL10 9AB, UK; 2 Expert at French Food, Environment and Work Safety Agency (ANSES), Former INSERM Senior Scientist, Cancer Institute, 34298 Montpellier cedex 5, France; E-Mail: mariette.gerber@sfr.fr

**Keywords:** Mediterranean diet, food processing, food preparation, cooking, phytochemicals, inflammation, oxidative stress

## Abstract

The benefits of the Mediterranean diet (MD) for protecting against chronic disorders such as cardiovascular disease are usually attributed to high consumption of certain food groups such as vegetables, and low consumption of other food groups such as meat. The influence of food processing techniques such as food preparation and cooking on the nutrient composition and nutritional value of these foods is not generally taken into consideration. In this narrative review, we consider the mechanistic and epidemiological evidence that food processing influences phytochemicals in selected food groups in the MD (olives, olive oil, vegetables and nuts), and that this influences the protective effects of these foods against chronic diseases associated with inflammation. We also examine how the pro-inflammatory properties of meat consumption can be modified by Mediterranean cuisine. We conclude by discussing whether food processing should be given greater consideration, both when recommending a MD to the consumer and when evaluating its health properties.

## 1. Introduction

Food preparation and cooking influence the nutritional qualities of foods, and potentially their health benefits. These processes can have beneficial effects, for example by improving the digestibility and bioavailability of nutrients, and by enhancing attractiveness to the consumer because of improved texture and taste [1], and also deleterious effects due to loss of nutrients or the formation of toxic compounds [2]. The Mediterranean diet (MD) is well-known for its health benefits and this diet is increasingly being recommended in non-Mediterranean countries [3]. However, food preparation and cooking customs in non-Mediterranean countries may be quite different from those in Mediterranean countries. This is rarely considered when applying findings on the MD derived from one population to a different population with a different cuisine, and the extent to which differences in food preparation practices between populations impacts on the nutritional qualities of the MD is not well understood.

The traditional MD is characterized by plenty of fruits, vegetables and nuts, legumes, cereals (preferably whole grain), herbs and spices, fish and seafood, moderate amounts of meat and dairy produce (mainly from sheep and goats), olive oil (for dressing and cooking) and moderate amounts of wine with meals [4]. This rich diversity of foods results in the MD having a unique compendium of nutrients that contributes to its protective effects against chronic disease [5,6]. Because the MD is essentially a plant-based diet, phytochemicals are considered to be major contributors to the overall health benefits of this diet [7], and this group of nutrients is strongly influenced by food preparation and cooking [8,9]. A major property of phytochemicals that contributes to their health benefits is their ability to reduce oxidative stress and inflammation (reviewed in [10]), and a reduction in inflammation by phytochemicals has been linked to the benefits of phytochemicals for the MD [11].

In this review, we first consider the anti-inflammatory effects of the MD, and we then go on to discuss antioxidant and anti-inflammatory phytochemicals in some of the main food groups of the MD (olives and olive oil, vegetables and nuts). We then discuss how these phytochemicals are influenced by food preparation and cooking and the effects of these processes on health outcomes. Since the overall oxidative stress and inflammation in the body is due to factors that increase as well as decrease oxidative stress and inflammation, we also consider food preparation and cooking procedures that are pro-oxidant and pro-inflammatory, particularly in relation to meat. Because some traditional Mediterranean cooking practices may be less common in non-Mediterranean countries, we conclude by discussing whether advice on food preparation should be given when recommending the MD in non-Mediterranean countries.

## 2. Oxidation, Inflammation and the MD

Inflammation is now recognized as a major factor in the pathology of many chronic diseases including cardiovascular disease (CVD), cancer, type 2 diabetes, metabolic syndrome and Alzheimer’s disease, and inflammation is also associated with obesity [10,12,13,14,15]. The inflammation that contributes to these diseases may occur systemically in the body, such as the low grade chronic inflammation that is also linked with aging (inflammaging) and obesity, or it may be more localized. Another source of inflammation with potentially harmful consequences is the acute inflammation that can occur during the postprandial state as a result of hyperlipemia and hyperglycemia [16].

A number of epidemiological studies have demonstrated that consuming a MD reduces inflammation [14]. Barbaresko and colleagues in a recent systematic review reported that among *a priori* healthy dietary patterns, the MD was the most consistent in showing a decrease in C reactive protein (CRP) and an increase in adiponectin [17]. In the large Italian Moli-sani study, a decrease in white blood cell and platelet counts (measured as markers of low grade inflammation) was shown to be associated with a greater adherence to a Mediterranean-like diet [18]. Adherence to a traditional MD was also shown to be associated with a reduction of CRP and IL-6 in survivors of a myocardial infarct, independently of the medication [19]. The potential role of the MD in reducing low grade chronic inflammation in the elderly (inflammaging) is currently being studied in the NU-AGE project [20].

As well as low grade inflammation, there is also good evidence that consuming a MD reduces postprandial inflammation [21,22,23], and the postprandial oxidative stress that can result from postprandial hyperglycemia and hyperlipemia [23]. One link between oxidative stress and inflammation is through the transcription factors NF-κB. This is because NF-κB is induced by oxidative stress, which in turn increases the expression of pro-inflammatory genes for cytokines and chemokines [24]. Hence, not surprisingly, antioxidant phytochemicals can reduce inflammatory responses. For example, in a short term intervention it was shown that polyphenols from virgin olive oil (VOO) decreased postprandial gene expression of the NF-κB-mediated pro-inflammatory cytokines *IL1B*, *IL6* and *CXCL1* in peripheral blood mononuclear cells [22]. In the Predimed (Prevención con Dieta Mediterránea) study, adherence to a MD was associated with increased total antioxidant capacity (TAC) [25], and high dietary anti-oxidant levels lowered CRP and IL-6 [26]. Total dietary antioxidants independently explained the relationship between adherence to a MD pattern with better health-related quality of life (especially mental rather than physical health) in the Moli-sani study [27].

Many of the nutrients, such as saturated fats, *trans* fats and refined carbohydrates, that are associated in *in vitro* models with inflammatory responses are present at far lower levels in the MD than are typically found in the western diet [28]. In order to quantify the overall inflammatory potential of dietary patterns, a Dietary Inflammatory Index (DII) has been developed [29]. The DII is computed from the inflammatory activity of 45 foods and nutrients. Some studies suggest that DII is positively associated with an increased risk of various diseases such as pancreatic cancer [30] and colon cancer [31]. Using data from the Predimed study, a DII was computed from a measure of adherence to the MD (a MD score) and, as expected, the MD score was found to be inversely associated with DII [32]. In this cross-sectional analysis of the Predimed study, a lower DII was associated with a lower prevalence of obesity [32] and in a prospective one with incidence of CVD [33]. Based on nutritional data from Luxembourg, higher adherence to a MD score was associated with a favorable cardio-metabolic, hepatic and renal risk profile, whereas the relationship with DII was weaker suggesting that other foods and nutrients than the ones used in the construction of the DII are necessary for greater prevention [34]. Hence, in summary, it can be concluded that there is good evidence that antioxidant phytochemicals in the MD have anti-inflammatory effects in the body, and there is increasing evidence that this is important for the health benefits of the MD.

## 3. Food Processing and Phytochemicals—General Considerations

When establishing associations between adherence to the MD and the inflammatory status of the diet (e.g., using DII), the nutritional data are based on food composition tables that usually do not take into consideration possible effects of food preparation and cooking. Loss of anti-oxidant phytochemicals can occur during cooking due to thermal degradation and from leaching of substances (especially of more polar compounds) into the cooking medium. As well as loss of antioxidants, the formation of pro-oxidants can occur, especially when cooking at high temperatures, notably as a consequence of the Maillard reaction. Food preparation can also have beneficial effects by increasing the bioavailability of antioxidant phytochemicals [35]. For some phytochemicals, this is because of enhanced release from the food matrix, although the extent to which this occurs may vary widely. Carotenoids can be released from their association with proteins inside chromoplasts, and folate from proteins [36,37]. However, some polyphenols such as phenolic acids are more tightly bound to dietary fiber and protein [38]. Bound polyphenols can constitute a significant proportion of total daily intake. In fruits (apples, peaches and nectarines), 80%–90% of polyphenols were in the bound form [39], and it was estimated that bound polyphenols constituted 78% of the total phenolics in the Spanish diet [40]. Hence, factors that release bound polyphenols may have a significant impact on the overall level of bioavailable polyphenols. Poor bioavailability is not, however, necessarily detrimental. Components of the food matrix can act as carriers enabling phytochemicals to reach the colon where they may be released by the actions of gut bacteria. For example, grain fiber carries polyphenols to the colon and releasing polyphenols here, rather than higher up the gastrointestinal tract, may have health benefits either in the colon itself or after being absorbed there into the body [41,42].

Because it is difficult to predict the consequences on health of food processing simply based on model systems, we consider a wide range of studies (biochemical studies, biomarkers and health outcomes) that address how food processing may influence both the bioavailability and health consequences of consuming antioxidant and anti-inflammatory phytochemicals present in the main foods groups of the MD.

## 4. Olive Oil and Olives

Antioxidant and anti-inflammatory compounds in extra virgin olive oil (EVOO) include tocopherols, carotenoids and polyphenols. Polyphenols are especially important because of their number, diversity and particular properties. At least 36 phenolic compounds have so far been identified in EVOO [43]. The most abundant polyphenols in EVOO include secoiridoids such as oleuropein derivatives (especially the dialdehydic form of decarboxymethyl elenolic acid linked to hydroxytyrosol), phenolic derivatives of secoiridoids such as tyrosol and hydroxytyrosol, lignans, flavonoids, and decarboxy methyl ligstroside aglycone—known as oleocanthal [44,45]. The antioxidant activities of EVOOs have been shown to correlate with their polyphenol content [46,47], one study finding a better correlation with diphenol content than with overall phenol content [47]. Ortho-diphenols such as hydroxytyrosol and oleuropein derivatives are more potent phenolic antioxidants [46,48]. Oleocanthal is an inhibitor of cyclooxygenase activity and is best known for its anti-inflammatory properties [44]. The major polyphenols hydroxytyrosol and tyrosol have high bioavailability (40%–95%) when present in olive oil [49], although there is less information for other EVOO polyphenols.

The health benefits of olive oil have been extensively reviewed [50,51,52]. When compared with olive oils with low polyphenol concentrations, most clinical studies demonstrated that olive oils containing high polyphenol concentrations resulted in greater reductions in inflammatory biomarkers, better protection of low-density lipoprotein-cholesterol (LDL-cholesterol) from oxidation, and greater decreases in isoprostanes [43]. EVOO has been shown to be superior to ordinary olive oil (which is lower in polyphenols) in preventing CVD [53]. Although there is suggestive evidence for a protective effect of olive oil against breast cancer [54], studies have not distinguished between ordinary olive oil and EVOO. The antioxidant properties of polyphenols are implicated in reducing CVD and some cancers (with levels of evidence categorized as possible to probable) by preventing LDL oxidation and neutralizing free radicals respectively [55]. The anti-inflammatory properties of polyphenols from EVOO may also contribute to these beneficial effects [56]. Oleocanthal has neuro-protective effects and attenuates markers of inflammation implicated in Alzheimer’s disease, and has also been shown to have anti-proliferative effects against human breast and prostate cancer cell lines [44,57].

Table olives are extremely rich sources of antioxidant polyphenols, comprising 1%–3% of the fresh pulp weight [48]. For most types of olives, the major polyphenols are oleuropein and hydroxytyrosol. In one study, levels of hydroxytyrosol were estimated to be 250–760 mg/kg in Kalamata olives, 170–510 mg/kg in Spanish-style green olives, and 100–340 mg/kg in Greek-style naturally black olives [58]. Thus, one Kalamata olive (4 g flesh) provides approx. 1–3 mg of hydroxytyrosol. In comparison, data from the Phenol-Explorer database gives an average content in EVOO of 7.7 mg hydroxytyrosol/kg oil [59]. Not surprisingly, olive extracts exhibit high antioxidant activity in *in vitro* assays [60,61]. Despite the high levels of hydroxytyrosol in table olives compared to EVOO, the bioavailability in humans of hydroxytyrosol and other polyphenols from table olives is currently unknown and to the best of our knowledge there are no human studies on their health effects, possibly because of their frequent high salt content (although this can be reduced by rinsing).

### 4.1. Effects of Processing

Concentrations of total polyphenols in olive oils vary considerably—between 50 to 800 mg/kg depending on the quality and type of the oil [62]. The extraction technique is generally considered to have the greatest impact; the first extraction at low temperatures produces virgin olive oil and this has the highest level of polyphenols. However, other factors also influence polyphenol levels including the geographical location, growing conditions and cultivar of the olive trees, ripeness of the olive at harvest, possible infestation with the olive fly, extraction method for the oil, and storage of the oil [46]. For example, a 13-fold difference in diphenol content was found between EVOOs from different cultivars from various regions in Italy [47].

Antioxidant levels in table olives also vary widely. A 10-fold or more difference was found in levels of hydroxytyrosol between olive cultivars [48]. The production method for the olives is also very influential. Particularly dramatic is the almost complete loss of hydroxytyrosol, due to oxidation and polymerization, during the California-style oxidation method for converting green olives into black olives [48,63]. Pitting of olives is also influential: an additional washing step is required when olives are pitted, and this process was found to almost half the content of hydroxytyrosol that had been present in the un-pitted olives [64].

In relation to olive oil, a major influence for the consumer is how the oil is used for cooking. Common domestic cooking conditions include shallow frying (which is at approx. 140–160 °C), in soups and stews (which may include an initial frying period, followed by boiling at 100 °C or at a higher temperature if the dish is cooked in the oven), and as an addition towards the end of cooking, for example in the “lathera” dishes from Greece and Turkey. In Portugal, vegetable soups with added olive oil are very popular, and in a study that mimicked domestic conditions there was found to be less loss of tyrosol and hydroxytyrosol from the oil and phenolics from the vegetables if the oil was added towards the end of the cooking period rather than at the beginning [65]. However, not all studies have replicated conditions likely to be encountered during normal domestic cooking.

When olive oil is used for frying, the stability of olive oil polyphenols is strongly influenced by cooking temperature and time, the type of food present and the composition of the oil (including antioxidants and polyunsaturated fatty acid (PUFA) content) [52,66]. In a study that simulated frying by heating olive oil (although in the absence of food) to 180 °C, there was a significant decrease in hydroxytyrosol derivatives (60% reduction after 30 min and 90% reduction after 60 min) [67]. Similar results were reported for the mass-produced Bertolli EVOO [68]. A study from Spain found far higher loss of hydroxytyrosol derivatives from oils made from Arbequina olives than for Piqual olives [69], and it has been suggested that the higher rates of loss may reflect a higher PUFA (linoleic acid) content of oils derived from Arbequina olives [66].

In contrast to the secoiridoids, lignans and oleocanthal are relatively heat stable [69]. For example, 12 frying cycles of 10 min each at 180 °C only reduced oleocanthal levels by 20% [70], although the degree of loss may depend on the initial concentration of oleocanthal in the oil [71].

In a short term human study that compared the effects of heated oils on the postprandial inflammatory response in obese subjects, VOO repeatedly heated to 180 °C was found to suppress postprandial inflammation in obese subjects (determined as NF-κB activation in peripheral blood monocytes) [72]. Hydroxytyrosol was completely depleted by the heating process and so cannot be responsible for the observed effect. By contrast, some other polyphenols were preserved, including about 75% of tyrosol and 20% of oleuropein aglycones. Further work is required to establish the mechanism for the protective effect. Other than this study, most intervention studies in humans with olive oil have either used raw olive oil, such as EUROLIVE (the effect of olive oil consumption on oxidative damage in European countries) with oxidized LDL-cholesterol as an endpoint, or have not specified the use of the oil, e.g., Predimed with cardiovascular death as the primary endpoint. Moreover, none of the large number of prospective epidemiological studies examining the health benefits of consuming olive oil have distinguished between the consumption of raw and cooked olive oil. A recent systematic review concluded that there is no epidemiological evidence that consuming fried foods is associated with an increased risk of cardiovascular disease, although there was some evidence for weight gain [73]. Many of the studies used olive oil as the frying oil, but, as the authors point out, the precise outcome is likely to be influenced by the type of oil, frying technique (shallow frying or deep frying), frying duration and temperature, and use of new or reused oils.

Acrylamide has been found in black olives prepared by the California-style processing method [74]. Acrylamide is classified as a probable human carcinogen by the International Agency for Research on Cancer. Concentrations of acrylamide in California-style black olives varied between 410–512 μg/kg in this study and these concentrations are comparable to levels of acrylamide found in French fries, a better known dietary source of acrylamide. The acrylamide appears to be generated during the sterilization process of the olives [74]. By comparison, in this study, Spanish olives and Greek olives were found to have very low levels of acrylamide (<1.4 μg/kg). This is probably because these olives were not sterilized, and this is the normal case with black olives from some countries.

### 4.2. Implications

The European Food Safety Authority (EFSA) recently upheld a health claim that EVOO reduces LDL oxidation if “a minimum of 5 mg of hydroxytyrosol and its derivatives is consumed per day” [75]. However, because information on the polyphenol content of EVOOs is not at present commonly available to consumers, they are unlikely to know whether or not they have attained the goal recommended by EFSA. Labeling bottles of olive oil with their content of secoiridoids and making consumers aware of the possible losses of hydroxytyrosol due to high temperature cooking would facilitate implementing the EFSA guideline without requiring excessive intake of olive oil. There may already be some public understanding of possible losses of active compounds in olive oil: Portuguese consumers are apparently aware that adding olive oil towards the end of cooking is more beneficial [65]. As well as recommending EVOO for cooking, the use of raw EVOO could also be encouraged.

Due to the absence of human studies, there are currently no guidelines from EFSA recommending olives as a source of hydroxytyrosol and its derivatives. However the high levels of these compounds in certain types of olives, and because olives are mostly eaten raw, suggests that consumption of only a few olives could achieve the EFSA recommended levels and without consuming many calories (assuming there is good bioavailability). However, olives have markedly different levels of hydroxytyrosol and hence specific types of olives would need to be recommended. In particular, California-style black olives are not comparable to many other types of olives because of their low levels of hydroxytyrosol and quite high levels of acrylamide.

Because of the good evidence that polyphenols in EVOO contribute to its health benefits, it has been suggested that EVOO should be specifically recommended as part of a MD, rather than a more general recommendation of “olive oil” [76]. Although concentrations of hydroxytyrosol will vary depending on whether EVOO is consumed raw or after frying, how EVOO is used is not currently incorporated into estimates of consumption for calculating MD scores. Upper *versus* lower quantiles of olive oil intake in health studies mostly vary between 2–3 fold (see [52]). This variation is lower than the potential influence of food processing practices on hydroxytyrosol levels. Hence, variation in usage of olive oil is a potential confounding factor when evaluating the link between its consumption and health benefits. However, it should be noted that several other olive oil polyphenols are more stable than hydroxytyrosol.

## 5. Vegetables—General

Vegetables contain a wide range of antioxidant phytochemicals. The levels of these phytochemicals are influenced by environmental conditions such as exposure to sunshine, and by agronomic practices such as the choice of cultivar, use of fertilizers and whether the produce is grown organically or conventionally (see [77,78,79] for recent reviews). Epidemiological studies have compared the health benefits of raw and cooked vegetables, and this is particularly relevant to the MD since consumption of raw vegetables as a proportion of total vegetable consumption was reported to be higher in southern cohorts from the EPIC (European Prospective Investigation into Cancer and Nutrition) study compared to their northern counterparts [80]. In a recent review of case-control studies from Southern European countries, both raw and cooked vegetables protected against various cancers, especially upper digestive tract cancers, but there was greater protection with raw vegetable consumption [81]. In the case of breast cancer, only raw was beneficial [81]. However, since this analysis was based on case-control studies, it is possible that cases may have modified their intakes. In a large prospective study with a follow-up > 10 years, raw vegetables was the only category of fruit and vegetables that was significantly and strongly (50%) associated with a risk reduction for stroke [82].

Salads are a significant source of raw vegetables in the MD. In an analysis of the traditional Cretan Mediterranean diet, salads were found to be consumed several times per week [83]. This way of eating not only preserves heat labile nutrients, but dressing salads with olive oil, vinegar, herbs or spices can greatly increase the antioxidant capacity of the dish. For example, the herbs lemon balm and marjoram (1.5% w/w) increased the antioxidant capacity of a salad by 150% and 200% respectively [84]. Many herbs are consumed in high amounts in the MD compared to a typical western diet, and they are frequently consumed raw or with minimal cooking. Herbs are particularly high in antioxidant and anti-inflammatory compounds, especially polyphenols [84,85]. Herbs and spices contributed significantly to the overall dietary intake of flavonols and flavones of traditional Greek cuisine, and this was linked to their frequent consumption despite only being used in small quantities [86]. There is increasing recognition of the importance of herbs and spices for good health [87,88], and although herbs and spices are recommended foods in the MD, they are not currently assessed when measuring adherence to the MD.

Cooking processes can strongly influence antioxidant phytochemical levels in fruits vegetables and herbs [84,89]. Amongst the many ways of preparing vegetables, perhaps the most frequent and traditional way in Mediterranean cuisine is to present the vegetables in olive oil, either by cooking the vegetables in olive oil or by dressing the vegetables with raw oil. Olive oil enhances taste and acceptability and, it has been proposed, is an important reason for the high levels of consumption of vegetables in Mediterranean countries [90]. Another beneficial effect of EVOO is that when vegetables are cooked in this medium they may absorb significant levels of antioxidants from the oil [91]. In addition, the bioavailability and absorption of many phytochemicals in vegetables, especially carotenoids, is significantly enhanced and this may increase the health benefits. We now consider how food processing may influence the nutritional value of vegetables pertinent to the MD.

## 6. Tomato and Other Carotenoid-Containing Vegetables

The six main carotenoids usually reported to be present in human blood plasma are α-carotene, β-carotene, lycopene, β-cryptoxanthin, zeaxanthin and lutein. In the MD, these are obtained from a wide range of green leafy and other types of vegetables, fruits, cereals and olives [92]. Other potentially significant, but usually overlooked, carotenoids in the MD are the red pigment capsanthin and the yellow carotenoids crocin and crocetin. Capsanthin is the main carotenoid in red peppers and paprika, comprising 29.2–36.2 μg/g fresh weight in red pepper [93]. This compares with levels of lycopene—the main carotenoid in tomato—of 8.5–127.0 μg/g fresh weight [94]. The health significance of capsanthin is not known, and it does have a rather short half life in human plasma (20 h) compared to that of lycopene (222 h) [95]. Crocin and crocetin are the two main carotenoids in saffron and have anti-tumor effects in *in vitro* and *in vivo* models [96]. Carotenoids are highly reactive towards oxygen and free radicals and have antioxidant and anti-inflammatory effects in the body [97]. Lycopene also suppresses carcinogenesis by inducing apoptosis and inhibiting proliferation and metastasis, and by inducing cyto-protective enzymes [98].

### 6.1. Food Processing

In many green leafy vegetables carotenoids are associated with proteins, whereas in carrots and tomatoes carotenoids exist in a semi-crystalline form. Chopping, blending and cooking help release carotenoids from the food matrix. Subsequently, carotenoids must be incorporated into lipid micelles in the gut lumen in order to be absorbed across the gut wall. The effects of cooking techniques on levels of carotenoids was evaluated in a meta-analysis by Murador and colleagues [99]. From the pooled data, stewing (a widely used technique in Mediterranean cuisine) increased overall carotenoid content by 36%. The apparent increase in carotenoid concentrations during stewing may be due to a combination of little thermal degradation and high water loss. Other techniques either had no effect or decreased levels—by 41% in the case of frying.

Whereas chopping and the use of sauces are widely used in different national cuisines for carotenoid-containing vegetables, especially tomatoes, it is the predominance of olive oil as the main dietary fat that characterizes the traditional MD. There is evidence from *in vitro* and animal studies of carotenoid micelle formation and uptake that olive oil enhances the bioavailability of carotenoids to a greater extent that some other fats [100]. In particular, long chain fatty acids are superior to medium chain fatty acids and oleic acid is superior to PUFAs (linoleic acid) [100]. There was greater bioavailability of carotenoids (lycopene and astaxanthin) in rats given oral doses of carotenoids in olive oil compared to corn oil [101] and higher levels of the photo-protective carotenoids lutein and zeaxanthin were found in the eyes of rats given these carotenoids in an emulsion with olive oil compared to an emulsion of sunflower oil or groundnut oil [102].

Despite these observations, their significance for humans is unclear since the amount of lipid in the meal may be a more significant factor for carotenoid bioavailability than the actual lipid composition [103]. It has been estimated that 3–5 g of dietary fat is required in order to significantly enhance the bioavailability of dietary carotenoids [104,105]. Certainly, excluding lipid altogether can significantly reduce carotenoid uptake. For example, cooking diced tomatoes in EVOO increased plasma concentrations of *cis*-lycopene (the more biologically active isomer [106]) and *trans*-lycopene by 40% and 82% respectively, whereas cooking the tomatoes without EVOO increased plasma concentrations of *cis*-lycopene by only 15% and there was no increase in *trans*-lycopene levels [107]. Similarly, refined olive oil enhanced the absorption of *cis* and *trans* isomers of lycopene (as determined by plasma levels and AUC) in tomato juice in healthy volunteers compared to when the oil was absent [108].

Olive oil has also been shown to enhance the bioavailability of more polar phenolic compounds. For example, the extraction of phenolic compounds such as chlorogenic acid and naringenin (the main polyphenol in tomatoes) from a tomato sauce were enhanced when the tomato sauce was prepared with VOO [109]. In a cross-over study, there was some evidence of higher bioavailability of naringenin when human subjects were given a tomato sauce prepared with VOO compared to when they were given a tomato sauce prepared in the absence of VOO [110]. However, in a prospective randomized, cross-over intervention study, mechanical and thermal treatments during tomato sauce processing enhanced the bioavailability of various phenolics including naringenin to a greater extent than added lipid (refined olive oil) [111] Hence, added lipid may be less important for phenolic bioavailability from tomatoes compared with lycopene bioavailability.

A number of studies have also examined if processing tomatoes affects disease outcomes, although it is not usually specified if the consumption of the tomatoes also included olive oil consumption. A Greek case-control study examined the relationship between tomato consumption and risk of prostate cancer. Olive oil is likely to have been the main dietary fat in this Mediterranean population. Intake of either raw or cooked tomatoes reduced prostate cancer risk, although there was a significant and greater risk reduction for cooked tomatoes (OR lowest *versus* highest tertile 1.91; CI 1.20–3.04 for cooked, *versus* OR 1.55; CI 1.00–2.52 for raw) [112]. Chen *et al.* recently conducted a meta-analysis of prospective studies of prostate cancer incidence and tomato consumption but there were too few studies on raw and cooked tomatoes (3 studies in each category) to draw firm conclusions regarding the effects of processing [113]. The lipid content of the meals was not known and so could be a confounding factor in the analysis of these data. The roles of various phytochemicals in tomato as preventive agents against prostate cancer and the importance of other dietary constituents for increasing the bioavailability of these phytochemicals also requires clarification [114]. However, in the meta-analysis conducted by Chen *et al.* there were enough studies with no heterogeneity to show that there was no association between lycopene and prostate cancer incidence [113].

Bioavailability of carotenoids is modified by factors besides food processing such as the adiposity of the subject, and exposure to tobacco or air pollution. This is an important aspect when assessing risk reduction of cancers through dietary questionnaires, and, since these factors are not always taken into consideration, this might explain some of the inconsistencies observed in epidemiological studies. Studies using biomarkers show stronger and more significant associations between plasma carotenoids and cancer risk than if only dietary intake of carotenoids is assessed. This has been shown clearly for the association with breast cancer [115,116]. It is also noteworthy that the enzymes that metabolize lycopene are polymorphic. This contributes to wide inter-individual variations in lycopene plasma levels and so will influence health outcomes potentially associated with this carotenoid [106].

In relation to CVD, a short term study found that enhancing carotenoid bioavailability favorably influenced biomarkers for CVD as end points. In a small (11 subjects) randomized cross-over trial, LDL cholesterol and total cholesterol decreased significantly after the consumption of tomato with olive oil, and this was associated with an increase of *trans*-lycopene and 5-*cis*-lycopene, respectively [108].

### 6.2. Implications

Food processing and the presence of fat increase carotenoid bioavailability and there is some limited evidence that increasing carotenoid bioavailability may improve health benefits, at least in relation to CVD. It is less clear if preparing the food with fat is superior to simply having fat in the meal. Also, more work is needed to clarify if olive oil has benefits over other types of oils in relation to carotenoid absorption. Nevertheless, EVOO does have the added advantage that when vegetables are fried in it they may absorb beneficial micronutrients from the oil.

## 7. Cruciferous Vegetables

Cruciferous vegetables (e.g., cabbages, broccoli, cauliflower), also known as brassicas, contain a wide range of health promoting compounds including polyphenols, carotenoids, tocopherols and vitamin C. However, it is the presence of glucosinolates (GSLs) in crucifers that distinguishes them from other vegetables, and it is these compounds that have received the most attention. Most GSLs are considered to be inactive, but when they come into contact with the enzyme myrosinase GSLs are converted into various bioactive products including isothiocyanates and nitriles.

Sulforaphane, the breakdown product of the GSL glucoraphanin, is found in a wide range of crucifers. Sulforaphane has anti-carcinogenic and cyto-protective effects through its ability to activate the transcription factor nuclear factor erythroid 2-related factor 2 (Nrf-2) that in turn induces phase 2 metabolizing enzymes and antioxidant proteins [117,118]. In animal models, sulforaphane protects not only against carcinogenesis but also against organ damage and various neurodegenerative diseases [117,119]. A number of clinical studies have shown an effect of supplementation with various crucifers or glucosinolates on factors implicated in carcinogenesis such as cytoprotective enzymes (e.g., glutathione transferase) and markers of *Helicobacter pylori* colonization and gastric inflammation [118]. Some prospective studies on breast cancer showed a risk reduction associated with crucifer consumption [120,121], and also some evidence for a risk reduction of for gastric cancer, although the evidence is limited [122].

### 7.1. Food Processing

Because of their pungent taste, most crucifers are cooked prior to consumption. Two major cooking processes that reduce GSL breakdown products are leaching of the water-soluble GSLs into cooking water and heat inactivation of myrosinase. McNaughton and Marks estimated that on average 36% of GSLs are lost following processing of crucifers [123]. However, actual losses will vary widely depending on the precise cooking technique employed. Leaching of GSLs during boiling in water, the most common cooking technique for crucifers in western countries, is estimated to lead to losses of between 25%–75% of GSLs [124]. It is therefore noteworthy that in Mediterranean cuisine crucifer leaves are commonly consumed as part of composite dishes in which the cooking liquor is also consumed (soups and stews).

Myrosinase is progressively inactivated by heat, hence reducing its capacity to generate bioactive GSL breakdown products. Consuming crucifers raw will not only ensure that myrosinase remains active but also avoid losses from leaching. Rocket (arugula) is the most common crucifer leaf consumed in salads. In Mediterranean countries, both the cultivated form *Eruca sativa* and the wild form *Diplotaxis tenuifolia* are consumed. Extracts of *E. sativa* reduced benzo(a)pyrene-induced genotoxicity against human hepatoma (HepG2) cells [125] and had a range of cancer chemopreventive effects in cell culture and animal models [126]. An isothiocyanate-rich extract of *E. sativa* was found to inhibit melanoma growth and angiogenesis in mice [127]. A wide range of glucosinolates has been identified in *E. sativa* and *D. tenuifolia* [128]. Unlike other glucosinolates, glucoerucin, the main glucosinolate found in *Eruca* species, has direct antioxidant activity as well as acting as a precursor for the isothiosulphanate erucin [129].

### 7.2. Implications

Since food processing strongly influences the levels of GSL breakdown products in cruciferous vegetables this may impact on the health benefits of consuming these vegetables. This is suggested in a case-control study that found that consuming raw crucifers was associated with a risk reduction of bladder cancer (OR 0.64; 95% CI 0.42–0.97) whereas this was not observed for total crucifer consumption [130]. Apart from plant myrosinase, GSL breakdown products are also generated by the action of gut bacteria. However, this is less efficient than by plant myrosinase [131]. Moreover, relying on this as a means of generating GSL breakdown products is also less desirable because of the wide variations between individuals in their capacity to breakdown GSLs; this has been attributed to inter-individual variations in gut microflora composition [132].

Preparation techniques that retain cooking liquid (e.g., soups and stews) or that do not inactivate myrosinase (e.g., consuming raw) will maximize available GSL breakdown products. Providing myrosinase in the form of crucifer leaves is one way to compensate for losses of myrosinase activity. Providing myrosinase in the form of a few raw crucifer leaves (broccoli sprouts) was found to enhance conversion of a glucoraphanin-rich powder [133]. In Italy and Provence, salads that include a few rocket leaves are popular and may be able to act in a similar way by enhancing the conversion of glucosinolates from a dish of cooked crucifers in which the myrosinase has been inactivated. Blanching prior to freezing is another process that inactivates myrosinase, and adding raw crucifer (0.25% daikon root) was able to restore 100% of sulforaphane formation in frozen broccoli [134]. Hence, a few raw crucifer leaves may also be useful when consuming frozen crucifers.

Although retaining GSLs might be desirable from a health point of view, GSL breakdown products are responsible for the pungent taste of crucifers and hence many cultures prefer to consume these vegetables cooked, a process which will reduce pungency but also GSLs. Both taste and texture affect the cooking time chosen by consumers and the impact of these decisions on GSL content has been modeled for broccoli consumption. This study found that the most common boiling time compatible with optimal sensory acceptability (20 min) corresponded to a 20% to 80% reduction in GSL content (depending on the amount of water added) [135]. In contrast to this usual convention in western cuisine of consuming crucifers as a separate vegetable, crucifers in Mediterranean cuisine are frequently consumed as part of composite dishes such as stews or soups. Here the taste of GSL breakdown products is complemented by other flavors and higher levels of GSLs in the dish may be more acceptable. In the case of raw rocket leaves where levels of GSL breakdown products cannot be manipulated by cooking, a sweetened salad dressing may be added, although in a sensory analysis by a group of panelists the bitterness of rocket was perceived as a positive attribute in itself [136]. Another source of GSLs in the traditional MD is wild greens. The collection and consumption of wild greens remains an important part of the traditional Mediterranean diet [137], and wild greens belonging to the crucifer family, such as wild mustards, will add to the overall dietary intake of GSLs.

## 8. Alliums

The MD typically includes high consumption of onions and garlic. Two groups of phytochemicals in alliums that have received particular attention for their putative health benefits are organo-sulfur phytochemicals (thiosulfinates) and flavonoids. Thiosulfinates are volatile, unstable compounds generated by the enzyme alliinase from precursors (*S*-alk(en)yl-l-cysteine sulfoxides). In garlic, alliin is first converted by alliinase to allicin which then degrades to various compounds including ajoene, diallyl sulfides and vinyldithiins. These compounds are rapidly metabolized *in vivo* [138], and one metabolite of diallyl disulfide and diallyl trisulfide, hydrogen sulfide, is implicated in the vasoactive effects of garlic [139]. Allicin has *in vitro* anti-bacterial action against *H. pylori* which is incriminated in stomach cancer [140]. High levels of flavonoids are present in onions and garlic; onions contain particularly high levels of quercetin and kaempferol glucosides and garlic contains myricetin glucosides [141]. Both organo-sulfur compounds and quercetin have antioxidant and anti-inflammatory properties [142,143].

As is the case for many individual fruits and vegetables, epidemiological studies of all sites cancer risk and garlic intake are inconsistent because most cancer sites have specific modifying factors. The World Cancer Research Fund/American Institute for Cancer Research (WCRF/AICR) qualified the level of evidence for a causal relationship between allium vegetable consumption and prevention of stomach cancer and for colorectal cancer as probable [144]. For colorectal cancer, this was confirmed in the WCRF/AICR Systematic Literature Continuous Update Project (CUP) of 2011 [145]. These collective expertise reports have subsequently been confirmed by several studies. A recent review of Southern European case-control studies (mostly Italian cohorts) suggested that high intakes of onion and garlic decreased the risk for cancers of the oral cavity and pharynx, oesophagus, colorectum, larynx, endometrium, ovary and kidney (garlic only) [81]. In addition, in a case control study raw garlic consumption (two or more times per week) was inversely associated with lung cancer (OR 0.56; 95% CI 0.44–0.72) [146]. The authors speculated that this may have been because the volatile oil from raw garlic (which includes allicin and diallyl sulfides) is largely excreted in the lungs and hence may be able to exert a beneficial effect there. In another meta-analysis of mostly case-control studies, combined allium intake (garlic, onions and others) was significantly associated with reduced gastric cancer risk (OR 0.54; 95% CI 0.43–0.65) [147]. However, a recent meta-analysis found no overall protective effect of garlic consumption, even in the cohort studies which included garlic supplements [148]. There was considerable heterogeneity between studies and when only the case-control studies were analyzed (excluding the cohort studies and supplements) there was a 37% risk reduction (combined risk estimate = 0.63; 95% CI 0.48–0.82) in spite of significant heterogeneity in this sub-group analysis.

Garlic is also linked to reducing cardiovascular risk based on favorable effects *in vitro* and in animal studies in preventing vascular inflammation and reactive oxygen species (ROS) production, increasing nitric oxide, and inhibiting platelet aggregation. [149]. However, only modest effects on blood pressure were described, and a recent Cochrane review of epidemiological studies found insufficient evidence to determine if garlic provides a therapeutic advantage *versus* placebo in terms of reducing the risk of mortality and cardiovascular morbidity in patients diagnosed with hypertension [150].

### 8.1. Food Processing

Although there is also some rationale from experimental studies that raw garlic may have beneficial effects over and above cooked garlic for the prevention of cancer and CVD, there are too few epidemiological studies to undertake this analysis [149,151]. Nevertheless, food processing may differentially affect flavonoids and organo-sulfur compounds since they have distinct properties. Leaching of water soluble compounds is less of an issue for onions and garlic since they usually form the base of a dish that is consumed in its entirety. However, onions and garlic are frequently subject to high temperature cooking by frying. After moderate frying (5 min), concentrations of quercetin glucosides (quercetin 4-glucoside and quercetin 3,4 di-glucoside) in onions increased even when expressed on a dry weight basis which may possibly be explained by loss of other soluble solids and/or increased extractability due to plant cell wall degradation [152]. Longer heat treatments (>10 min) decreased levels of the quercetin glucosides.

The alliinase system is analogous to myrosinase in crucifers by being activated by damage to the plant tissue and also by being susceptible to inactivation by heat. Crushing garlic and allowing time for allicin to accumulate before heating improved the *in vitro* antiplatelet activity of garlic compared to heating garlic without crushing. [153], and, similarly, conditions that retained thiosulfinate generation in onions, such as heating whole intact onions, enhanced their anti-platelet activity [154]. However, the relevance of these *in vitro* findings to *in vivo* is unclear because of the rapid metabolism of allicin that occurs *in vivo*.

### 8.2. Implications

Whereas moderate cooking may enhance the bioavailability of quercetin glucosides in onions, cooking is deleterious to the generation of organo-sulfur compounds. There is no evidence that alliin is converted by gut bacteria to allicin, hence food processing is an important way of generating bioactive organo-sulfur compounds. Although epidemiological evidence is currently lacking, evidence from experimental studies suggests that typical Mediterranean dishes that include raw garlic or raw onions may have greater protective effects than for heated garlic or onions where alliinase has been inactivated. Crushing garlic (common in Spain) rather than chopping, and finely processing onion (such as the custom of using grated onions in tagines) may also be beneficial by causing greater plant cell disruption which increases alliinase activity.

## 9. Nuts

Nuts are an important component of the MD and have been shown to have significant health benefits, especially against CVD and all-cause mortality (see [155,156] for recent meta-analyses). The Predimed study found that when nuts (a combination of walnuts, almonds and hazelnuts) were given to enrich a MD, they reduced the risk of incident CVD and of diabetes [157]. Data on cancer are currently too limited to draw conclusions [158].

Nuts contain broadly similar constituents, and most nuts are rich sources of unsaturated fats (and low in saturated fatty acids (SFA)), protein, arginine (a precursor of nitric oxide (NO), important for vascular tone), fiber and a variety of minerals and vitamins including tocopherols, folate, Mg and Ca. However, there are important differences between nuts. For example, although the predominant FA in most nuts is MUFA (oleic acid), walnuts contain only low levels of MUFA and are high in PUFA (linoleic acid and α-linolenic acid). α-Tocopherol predominates in almonds and hazelnuts but γ-tocopherol is the main form in walnuts and pistachios [159]. Nuts are also excellent sources of antioxidant phenolics, with some nuts having particularly high levels of proanthocyanidins and hydrolysable tannins (gallotannins and ellagitannins) (see [160] for recent review). The stilbene *trans*-resveratrol occurs in peanuts and resveratrol-3-β-glucoside (piceid) has recently been identified in almonds [161].

### 9.1. Food Processing

Two common processing techniques that may influence the health benefits of nuts are removal of the skins (pellicles) and roasting. The pellicle is a major source of “total antioxidant capacity” (TAC) as measured by the FRAP (ferric reducing antioxidant potential) assay, particularly in walnuts where, by one estimate, 95% of TAC was present in the pellicle [162]. In another study, removal of the pellicle reduced TAC by approximately 36% in hazelnuts, 90% in walnuts and 55% in pistachios [163]. This reduction in TAC can be explained because the pellicle is the location for the majority of phenolic antioxidants. For example, pistachio skins contain 70.27 mg of flavonoids/g fresh weight (expressed as catechin equivalents) whereas the kernel contains only 0.46 mg of catechin equivalents/g fresh weight [164]. Some nuts such as walnuts are almost always eaten with their pellicle whereas others, such as peanuts, are usually eaten without it. Peanuts are the main type of nut consumed in some non-Mediterranean countries, and so nut-derived antioxidant polyphenols may be lower in these countries. However, nuts without their skins still contain various fat soluble antioxidants such as tocopherols since these are present in the kernel [160].

Almond skin is an effective inhibitor of copper-induced oxidation of human LDL cholesterol [165]. However, the benefits of consuming the whole nut may be greater since antioxidant polyphenols from almond skins were shown synergise with α-tocopherol to increase the resistance of LDL to oxidation [166]. This may be explained by flavonoids recycling the radical generated by α-tocopherol during inhibition of LDL oxidation [167]. Beneficial effects have been observed even for nuts without their pellicles. For example, a recent crossover clinical study on 15 moderately hypercholesterolemic subjects examined the differential effects of whole walnut, walnut oil, meat and skin on biomarkers for cardiovascular disease risk [168]. Walnut oil favorably affected endothelial function, and whole walnuts increased cholesterol efflux.

Nuts are sometimes consumed after roasting (which is typically between 140–180 °C). Roasting may cause changes to nut lipids and phytochemicals and generate new products through the Maillard reaction. Some studies [169] but not others [170] found small increases in *trans*-FAs after roasting, although levels were not considered to be of a concern to health. Increases in lipid oxidation products (as measured by Thiobarbituric Acid Reactive Substances—TBARs) following roasting correlated with the PUFA content, and tocopherol levels were reduced by prolonged roasting [170]. Although roasting can reduce antioxidant polyphenols in the nut skins, prolonged roasting can increase overall TAC. Possible reasons for this have been suggested by Bullo and colleagues and include breakdown of polymeric polyphenols to monomers, hydrolysis of glycosylated flavonoids, the decomposition of aglycones and the formation of Maillard reaction products (MRPs) with antioxidant activity [171]. The Maillard reaction can also lead to the production of harmful products. One such product, acrylamide, is produced by roasting, especially in almonds at high temperatures, which is probably due to their high asparagine and reducing sugar content [172]. Advanced glycation endproducts (AGEs), which are mostly pro-inflammatory, also form when almonds [173] and other nuts [174] are roasted.

Although complex changes can occur to antioxidants in nuts during roasting, the effects of roasting on oxidative stress have not been examined in clinical studies to the best of the authors’ knowledge. A few clinical studies have examined the consequences of roasting on other health parameters. One study comparing the effects of unprocessed with processed peanuts (whole raw, roasted unsalted, roasted salted or honey roasted peanuts, or ground peanut butter) found that none of the forms of peanuts induced a change in body weight or lipid parameters (total, LDL-cholesterol and triglycerides) [175]. In a few hypercholesterolemic subjects, total cholesterol, LDL-cholesterol and triglyceride concentrations were reduced in the same way by unprocessed and by processed forms of peanuts. Similarly, both raw and roasted almonds lowered LDL-cholesterol and total cholesterol [176].

### 9.2. Implications

It is likely that the health benefits of nuts result from many components in nuts such as their healthy fat profile, high level of antioxidants, and high levels of arginine (which acts as a source of NO). *In vitro* studies suggest that whole nuts (with their skins) that are either unroasted or only moderately roasted will be optimal for health although clinical studies suggest that some lipid parameters are influenced similarly by roasted or unroasted nuts. Although assessments of nut intake in the MD do not distinguish between the type of nut, this could be a confounder, for example because of the significantly different fatty acid profile for walnuts. Retaining the skin also improves storage since the skin acts as a protective shield against oxidation of unsaturated fatty acids [177].

## 10. Meat

Low meat consumption is recommended as part of a MD. Red meat consumption was shown to increase inflammatory biomarkers in healthy women [178] and thus meat may work in opposition to the anti-inflammatory effects of many plant foods. Not only does a low meat diet reduce the DII [179], but aspects of Mediterranean cuisine may also contribute to reducing the inflammatory effects of meat as discussed below.

Red meat consumption is consistently associated in prospective studies with an increased risk of obesity [180,181,182,183] and diabetes [184,185,186,187,188,189,190]. For CVD, two recent meta-analyses showed an increased risk with red and processed meat, with a level of evidence of “probable” [191,192]. Cancers have also been associated with meat consumption particularly colorectal cancer. Red meat and processed meat have been analyzed separately, and the WCRF/AICR CUP qualified the level of evidence for a causal association between red meat and processed meat and colorectal cancer as convincing [145]. Since this study, six prospective studies [193,194,195,196,197,198], three meta-analyses [199,200,201] and one analysis of pooled studies [202] have shown some inconsistencies in relation to processed meat which might be related to different processing methods of meat between countries. However, the level of evidence for the association remains probable to convincing and underlines the importance of processing methods. Two prospective studies analyzed the relationship between prostate cancer and processed meat, and both showed a significantly increased risk suggesting a positive association [203,204].

One group of compounds implicated in the adverse effects of cooked meat is MRPs. Most MRPs in meat result from cooking at high temperatures, and although some MRPs contribute to the desirable flavor of cooked meat, others, including heterocyclic amines (HCAs) and advanced glycation endproducts (AGEs), are potentially harmful. HCAs are suspected carcinogens, whereas many AGEs have pro-inflammatory activities and are incriminated in a wide range of chronic diseases [205].

Although below median consumption of meat is positively scored for in the MD, there is no consideration of how cooking techniques, such as the degree of cooking or foodstuffs cooked with the meat, may modify the formation of pro-inflammatory or pro-carcinogenic MRPs. Here we briefly discuss the evidence for adverse health effects of MRPs in meat especially in relation to inflammation, and we then discuss if food processing techniques relevant to the MD may reduce their formation.

### 10.1. Heterocyclic Amines

Cooking meat at high temperatures generates a wide range of HCAs, and more than 25 have so far been identified [206]. PhIP (2-amino-1-methyl-6-phenylimidazo(4,5-b)pyridine) is the most abundant HCA detected in the human diet, followed by MeIQx (2-amino-3,8-dimethylimidazo(4,5-f)quinoxaline) and DiMeIQx (2-amino-3,4,8-trimethylimidazo(4,5-f)quinoxaline). In 1993 the International Agency for Research on Cancer concluded that the evidence from animal studies was sufficient to claim that PhIP and MeIQx are carcinogenic. Studies examining if HCAs contribute to the association between cooked meat and colorectal cancer risk are inconsistent (reviewed in [207]). The results of a case-control study strongly suggested an association with increased risk of breast cancer with high intake of well-done red meat and MelQx among postmenopausal women only (*P*-trend = 0.002 and 0.003, respectively) [208]. The evidence for a causal relationship between processed meat and breast cancer is limited and mainly observed in case-control studies where a genetic polymorphism in N-acetyltransferase 2 has been investigated in relation to the presence of HCA in foods (e.g., [209]). However, recent evidence does not suggest that polymorphisms in genes that metabolize HCAs (*N*-acetyltransferase 2 and CYP 1A12) modify colon or breast cancer risk [210,211]. In the National Institutes of Health (NIH)—AARP Diet and Health prospective study, there is suggestive evidence implicating HCAs found in red meat cooked at high temperatures in gastric cardia and non-cardia adenocarcinomas although the association never reached statistical significance [212].

### 10.2. Advanced Glycation Endproducts

AGEs result from dicarbonyl groups from carbohydrate degradation products reacting with an amino terminal group. Although AGEs are mainly products of the Maillard reaction, they can also be formed from the auto-oxidation of glucose or from the peroxidation of lipids into dicarbonyl groups due to oxidative stress [213,214]. As well as coming from the diet, AGEs are also produced endogenously in the body. Cooked meat is the major dietary source of AGEs in most countries, with fish, aged cheese and roasted nuts being other sources [215].

Many *in vitro* studies have shown that AGEs increase oxidative stress and inflammation and this may at least in part be mediated by binding of AGEs to a receptor (RAGE) [205]. A number of human studies have demonstrated a link between dietary intake of AGEs and inflammation, although results are not conclusive. From a recent systematic review of human intervention studies in both healthy individuals and patients with an underlying disease (mostly commonly type 2 diabetes), the authors concluded that the main effect was a decrease in inflammatory status on a low AGE diet, whereas results for an increase in inflammatory status on a high AGE diet were less consistent [216]. The response to AGEs may be related to the inflammatory status of the individual and the level of circulating AGEs [216]. AGEs have been implicated as causal factors for a wide range of diseases, in particular for type 2 diabetes [217,218] and also for memory decline and Alzheimer’s disease [219,220]. In a recent study, a high level of circulating methylglyoxal (MG)—an AGE precursor—was shown to correlate with impaired cognition in humans and impaired learning and memory function and enhanced amyloid-β accumulation in mice [221]. In addition, high dietary consumption of AGEs has been shown to be associated with pancreatic cancer [222]. Despite this accumulating evidence, the role of AGEs in disease pathology remains controversial and there are conflicting results both in preclinical and clinical studies, not least because of the lack of a fully validated assay for measuring the AGE content of foods [14,214].

There is some limited evidence linking AGEs with the MD. In a population of Italian students, loss of adherence to a MD correlated with an increased intake of AGEs [223]. A recent study that retrospectively estimated AGE intake from various ecological and cohort studies showed that high adherence to a MD resulted in a lower meat and dairy intake, both of which contained high levels of AGEs [215]. This study then compared the extrapolated AGE intake to Alzheimer’s disease incidence in two cohorts (Washington Heights-Inwood Community Aging Project cohorts from 1992 and 1999). They proposed a correlation between AGE intake and Alzheimer’s disease incidence although further studies are required to validate this study.

### 10.3. Food Processing

Several aspects of Mediterranean cuisine may contribute to reducing the inflammatory and carcinogenic effects of meat that are mediated by MRPs. The Maillard reaction is a non-enzymatic reaction that is influenced by a large range of factors. The reaction accelerates significantly at high temperatures, with a doubling in the rate with a 10 °C increase in temperature [224] and levels of AGEs in foods cooked at high temperatures are typically up to 100-fold above those found in uncooked foods [174]. This is an important consideration for meat since it is cooked at a wide range of temperatures ranging from 100 °C for poaching/stewing, 140–190 °C for frying, to over 200 °C for roasting and barbecuing. For example, levels of carboxymethyl lysine (CML) (an AGE precursor) determined by UPLC/MS were up to twice as high in roasted chicken compared to boiled chicken [225] The degree of “doneness” also influences AGE formation, and CML levels were twice as high in a well-done roasted beef joint compared to one roasted rare [225].

The Maillard reaction is low at acidic pH [214] and marinating beef with lemon juice or vinegar has been shown to reduce subsequent AGE formation during roasting [214]. Marinating is commonly used in traditional Mediterranean cooking to tenderize tough meat. Besides effects on pH, constituents in commonly used marinades have been found to reduce the formation of MRPs including some types of HCAs. In most cases, the reduction in HCA formation was attributed to the antioxidant activities of the marinades. For example, marinating chicken breasts with red wine reduced PhIP by up to 88% [226], marinating beef patties with garlic was especially effective at reducing the formation of MeIQx and 4,8-DiMeIQx [227], both MeIQx and PhIP were reduced by a rosemary extract [228]. VOO reduced the formation of various HCAs in fried burgers to a greater extent than non-VOO [229]. Various components in VOO may contribute to its ability to inhibit MRPs. Oleanolic acid—a main terpene in olive oil and especially in olives [230]—has anti-glycative effects in diabetic mice [231]. There is a good deal of interest in the development of oleanolic acid for the treatment of diabetes although multiple mechanisms may be involved in its action [232]. Olive phenolics, added as olive mill waste water, trap dicarbonyl compounds and are being studied as anti-glycative agents, but it is not known if VOO has similar properties to the olive mill waste water [233].

In Mediterranean cooking, meat is often cooked with other ingredients such as vegetables, fruits, herbs and spices. A wide range of these foodstuffs have been shown to inhibit MRP formation, including both HCAs [206] and AGEs [234]. For example, HCA formation in model systems comprising mixtures of sugars and amino acids or meat extracts was inhibited by carotenoids from tomatoes [235] and a cherry extract inhibited PhIP formation in beef patties by up to 93% [236]. However, not all studies have been positive, and no significant differences in HCA levels were found between meat samples cooked in the typical Portuguese way with garlic, wine, olive oil, onion, and tomato and in control meat samples (cooked without other ingredients) [237]. Many foodstuffs typically used in Mediterranean meat dishes including cinnamon, garlic and rosemary have been shown to inhibit the formation of AGEs in *in vitro* and animal models [234].

### 10.4. Implications

Not only is the MD low in foods with potentially high HCA and AGE contents—such as cooked meat, it is also high in foods with a low content of these MRPs—such as fish, grains, low-fat milk products, fruits, and vegetables. Lamb—the meat traditionally consumed most frequently in the MD—has low levels of AGEs compared to other meats [174]. The wide range of studies demonstrating that cooking meat with plant foods such as vegetables, fruit, herbs and spices lowers HCA/AGE formation, suggests that this could be a healthful attribute of Mediterranean cuisine in comparison to some other cuisines where meat is more typically cooked on its own. The overall composition of a dish, rather than any single factor such as cooking temperature, can be important for determining the formation of MRPs. Not only can many foodstuffs inhibit MRP formation but there may also be a negative impact of combining meat with sources of sugars. For example, fried battered/breaded cod (not a traditional technique in Mediterranean cuisine) had significantly higher CML levels than when the fish was grilled/roasted, and casseroled chicken had higher levels of CML than portions roasted on their own. This may be because reducing sugars in the prepared dish are reacting with lysine residues in the protein [225]. The consequences of heating food are complex and not all MRPs are equally harmful; indeed some are beneficial and some MRPs have antioxidant and anti-inflammatory properties [238]. MRPs from foods rich in protein and fat appear to be more damaging than MRPs from roasted coffee and the crust of bread. This may be related to the higher content of the AGE precursors CML and MG in foods rich in protein and fat [213].

Since the Maillard reaction generates products with favorable attributes such as color and aroma, the aim—for optimum consumer acceptability—should not be to eliminate the Maillard reaction altogether but rather to use cooking techniques that direct the reaction towards favorable products rather than towards harmful ones [214]. Further research is clearly required to elucidate the roles of HCAs and AGEs in health and their relation to food processing. The WCRF/AICR CUP in 2010 stated that “it is probable that HCAs found on the surface of well-done meat can cause colon cancer in people with genetic predisposition” but no recommendation was made to the general public [239]. In relation to AGEs, recommendations for reducing AGE formation are strongest for diabetic patients and those with kidney disease [214].

## 11. Discussion

The importance of considering the MD as more than just its food components is widely recognized. According to UNESCO’s broad definition, the MD includes “a set of skills, knowledge, rituals, symbols and traditions concerning crops, harvesting, fishing, animal husbandry, conservation, processing, cooking, and particularly the sharing and consumption of food” [240]. Other commentators have also emphasized the importance to the MD of practices such as food preparation methods and physical activity [241], eating patterns and the absence of, or type of, snacking between meals [242,243]. Recent updates of the MD pyramid also emphasize “the production, selection, processing and consumption of foods” and other aspects of the Mediterranean lifestyle [244] and this is summarized in Table 1. In this review, we provide further evidence of the importance of these wider considerations. In particular, we have identified several examples of food processing pertinent to the MD that can have a significant impact on the nutritional qualities of foods such as olives, olive oil and vegetables that are consumed as part of a MD.

**Table 1:** Selected examples of how food processing techniques influence the levels of phytochemicals in foods in the Mediterranean diet and levels of biomarkers.

Food	Food Processing Technique	Resulting Change			
		Phytochemicals & Other Compounds	Ref.	Biomarkers	Ref.
Olives	California-style Processing	↓ Hydroxytyrosol, ↑ acrylamide	[48,63]		
	Pitting	↓ Hydroxytyrosol	[64]		
Olive oil	Refining	↓ Polyphenols	[51]	↓ Protection of LDL-cholesterol from oxidation in humans	[51]
Extra virgin olive oil	Deep frying (heating to 180 °C)	↓ Hydroxytyrosol	[67]		
		Small ↓ in oleocanthal	[70]		
		Small ↓ in lignans	[69]		
		↓ Hydroxytyrosol, oleuropein aglycone, luteolin	[72]		
Tomatoes	Cooked in olive oil	↑ Lycopene bioavailability	[107,108]		
		↑ Naringenin bioavailability	[110]		
	Mechanical processing	↑ Naringenin bioavailability	[111]		
Crucifers	Boiling	↓ Glucosinolates	[124]		
	Chopping	↑ Isothiocyanates	[123]		
Onions	Frying 5 min	↑ Quercetin glucosides	[152]		
	Frying > 10 min	↓ Quercetin glucosides	[152]		
Garlic	Crushing	↑ Allicin	[153]	↑ *In vitro* antiplatelet activity	[153]
Nuts	Removal of pellicle	↓ Anti-oxidant polyphenols	[162,163,164]	↓ Total plasma antioxidant capacity in overweight and obese adults	[168]
	Roasting	↑ or ↓ Total antioxidant activity	[171]	No change in LDL- cholesterol lowering effect	[176]

Contrasting with this perspective, it is often considered that effectively promoting a MD in non-Mediterranean countries requires taking into consideration the cultural cuisine of the country in question, and allowing for the ways food items are typically prepared and cooked in the adopting country [3,245]. This potentially creates a mismatch between tailoring the MD to non-Mediterranean cuisines whilst at the same time respecting the specific cultural aspects of traditional Mediterranean cuisine. Fortunately, advice on food processing pertinent to the MD is, in most cases, still compatible with the cultural cuisine of consumers in non-Mediterranean countries, since it merely involves changing cooking practices (such as stewing rather than boiling vegetables) or selecting healthier variants of foods (e.g., of olives and olive oil). Further implications of food processing on the nutritional qualities of foods are discussed more fully in the sections of this review for each food group.

At present, there is only limited information regarding the extent to which changing food preparation and cooking practices influences levels of consumption of antioxidant and anti-inflammatory phytochemicals. However, significant steps in this direction have been taken with the development of a Retention Factor (RF) for polyphenols [246]. The RF is a measure of how levels of polyphenols in foods change with food processing, and RF values have been incorporated into recent estimates of polyphenol intake in cohorts from the EPIC study [247].

Further work is also required to establish if food processing influences health outcomes, and whether or not this information should be incorporated into measures of adherence to the MD. Adherence to the MD is usually determined by establishing a MD score for a population based on median consumption of various food groups and then grouping subjects into quantiles based on their score above or below the median value [248]. Upper *versus* lower quantile values typically vary 2–3 fold for olive oil (in populations where olive oil is commonly consumed) [52] and 2–3 fold for vegetable consumption [249]. This narrative review, and the more quantitative data from RF values from the Phenol Explorer database [9], indicate that different cooking methods can also give rise to several-fold differences in polyphenol content between vegetables, depending on the type of polyphenol and type of vegetable. Hence, food processing practices may have a significant impact on the nutritional quality of some foods in the MD, comparable in some circumstances to differences in intake between low and high consumers. The use of the RF database will help capture the influence of food processing on the nutritional values of some food groups within the MD. However, this database only applies to polyphenols and, as discussed in this review, there are many other influences on the nutritional value of food groups, and there are currently no databases that quantify these. In addition, there are practical difficulties in capturing a level of precision in epidemiological studies beyond “raw” and “cooked” for vegetables and “boiled” and “fried” for animal products, and this information is of limited value if it is not translated by food composition tables. In the same way, the use of herbs and spice might be evaluated by “yes” or “no” or, at the most, by frequency, but never quantitatively.

## 12. Conclusions

In this review, we have highlighted aspects of food processing pertinent to the MD that can influence the nutritional quality of foods, and we also provide evidence that this may impact on health outcomes, especially in relation to oxidative stress and inflammation. Currently, it is difficult to quantify this impact. Nevertheless, recognizing that food processing influences the nutritional quality of foods may be important for public health campaigns promoting the MD and for epidemiological studies comparing the health benefits of the MD between populations with different cooking practices.
